# Deteriorating microbiomes in agriculture - the unintended effects of pesticides on microbial life

**DOI:** 10.20517/mrr.2021.08

**Published:** 2022-01-25

**Authors:** Brendan A. Daisley, Anna M. Chernyshova, Graham J. Thompson, Emma Allen-Vercoe

**Affiliations:** ^1^Department of Biology, Western University, London, ON N6A 5C1, Canada.; ^2^Department of Molecular and Cellular Biology, University of Guelph, Guelph, ON N1G 2W1, Canada.

**Keywords:** Microbiome, host-microbe interactions, agriculture, pesticides, microbial evolution, microbe-xenobiotic interactions, bioremediation, environmental sustainability

## Abstract

There is emerging concern regarding the unintentional and often unrecognized antimicrobial properties of “non-antimicrobial” pesticides. This includes insecticides, herbicides, and fungicides commonly used in agriculture that are known to produce broad ranging, off-target effects on beneficial wildlife, even at seemingly non-toxic low dose exposures. Notably, these obscure adverse interactions may be related to host-associated microbiome damage occurring from antimicrobial effects, rather than the presumed toxic effects of pesticides on host tissue. Here, we critically review the literature on this topic as it pertains to the rhizosphere microbiome of crop plants and gut microbiome of pollinator insects (namely managed populations of the western honey bee, *Apis mellifera*), since both are frequent recipients of chronic pesticide exposure. Clear linkages between pesticide mode of action and host-specific microbiome functionalities are identified in relation to potential antimicrobial risks. For example, inherent differences in nitrogen metabolism of plant- and insect-associated microbiomes may dictate whether neonicotinoid-based insecticides ultimately exert antimicrobial activities or not. Several other context-dependent scenarios are discussed. In addition to direct effects (e.g., microbicidal action of the parent compound or breakdown metabolites), pesticides may indirectly alter the trajectory of host-microbiome coevolution in honey bees via modulation of social behaviours and the insect gut-brain axis - conceivably with consequences on plant-pollinator symbiosis as well. In summary, current evidence suggests: (1) immediate action is needed by regulatory authorities in amending safety assessments for “non-antimicrobial” pesticides; and (2) that the development of host-free microbiome model systems could be useful for rapidly screening pesticides against functionally distinct microbial catalogues of interest.

## INTRODUCTION

Arguably, there is a microbial component inherent to all known systems on Earth with cumulative evidence supporting that niche-adapted microbial communities play unequivocally important roles in total ecosystem functioning^[1]^. This includes, but is not limited to, the facilitation of marine and atmospheric biogeochemical processes, regulation of soil-plant nutrient cycling, and maintenance of healthy animal communities. Emerging ideologies such as “Planetary Health” and “OneHealth” emphasize these fundamental roles of microbial metabolic processes in supporting macroscopic reality at the systems-level, and further suggest that microorganisms should be viewed as unified constituents rather than as separate entities, as they have been historically regarded^[2,3]^.

Consistent with these schemas is the holobiont (or hologenome) theory of evolution^[4] ^which posits that host-microbe co-adaptation has driven functional interdependence between many animal species and their gut microbiomes (i.e., referring to the community of microorganisms residing in the intestinal tract as well as their collective metabolic potential). Exemplifying this interdependence, animal species frequently rely on their gut microbiomes for nutrition, pathogen exclusion, and immunomodulatory functions^[5]^. The human gut microbiome has been well characterized in this regard, although there is substantial evidence from insect species too - including the western honey bee (*Apis mellifera*). In particular, this eusocial insect species relies heavily on the bacterial members of its gut microbiota as a result of depauperate immune and detoxification gene repertoires^[6]^. Similar relationships exist between plant hosts and the microbe-dense soil zone surrounding plant roots, known as the rhizosphere (or “microbe storehouse”), that plays a multifunctional role in supporting plant growth and is a critical factor influencing crop yields in agriculture^[7]^. The functional similarities between the gut microbiome of animals and the rhizosphere microbiome of plants have been discussed previously^[8]^, as has the related theory of “the host microbiome as an ecosystem on a leash”^[9]^.

Here, we draw attention to the neglected fact that anthropogenic activities (primarily those over the past century relating to farming practices) have introduced an astonishing number of pesticides and other agrochemical xenobiotics into the environment (~90,000 active products registered in the NPIRS database alone^[10]^), and that many can exert unintentional antimicrobial activities that disrupt host-microbiome homeostasis^[11]^. These activities include the microbicidal or microbiostatic properties exhibited by various herbicides, insecticides, and fungicides, which together constitute over 95% of all pesticides used worldwide^[12]^. To note, these effects are often unforeseen (e.g., insecticides - by design - target insects, not microbes) and are not adequately monitored by regulatory agencies since most pesticides are classified as “non-antimicrobial” chemicals. It is thus conceivable that widespread extinction of plant and animal host-adapted microbes may already be occurring, undetected, as a result of chronic sub-lethal pesticide exposures (i.e., through the use of compounds deemed non-toxic to the physiology of off-target host species, but not necessarily their microbiomes). Nonetheless, it is difficult to ascertain the extent of damage, since baseline host-associated microbiome data is often lacking.

We can gain some insight into the long-term consequences of microbiome damage from industrialized human societies that have undergone a systematic depletion in host-adapted microbes due to transgenerational antibiotic exposure (i.e., missing microbe hypothesis^[13]^) and excessive use of disinfectants (i.e., hygiene hypothesis of disease^[14]^). Importantly, these reductions in microbial diversity are directly associated with altered functionality of the gut microbiome, and are thought to represent a major instigating factor behind the growing global epidemic of chronic, non-communicable, metabolic disease^[15]^. Such metabolic disorders include irritable bowel syndrome^[16]^, type-2 diabetes^[17]^, obesity^[18]^, atherosclerosis^[19]^, and several types of cancer^[20]^. Thus, while the use of antibiotics and disinfectants have undoubtedly revolutionized clinical healthcare and tremendously reduced the spread and lethality of infectious diseases, persistent exposure to antimicrobial agents may pose significant long-term health complications.

**Figure 1:**
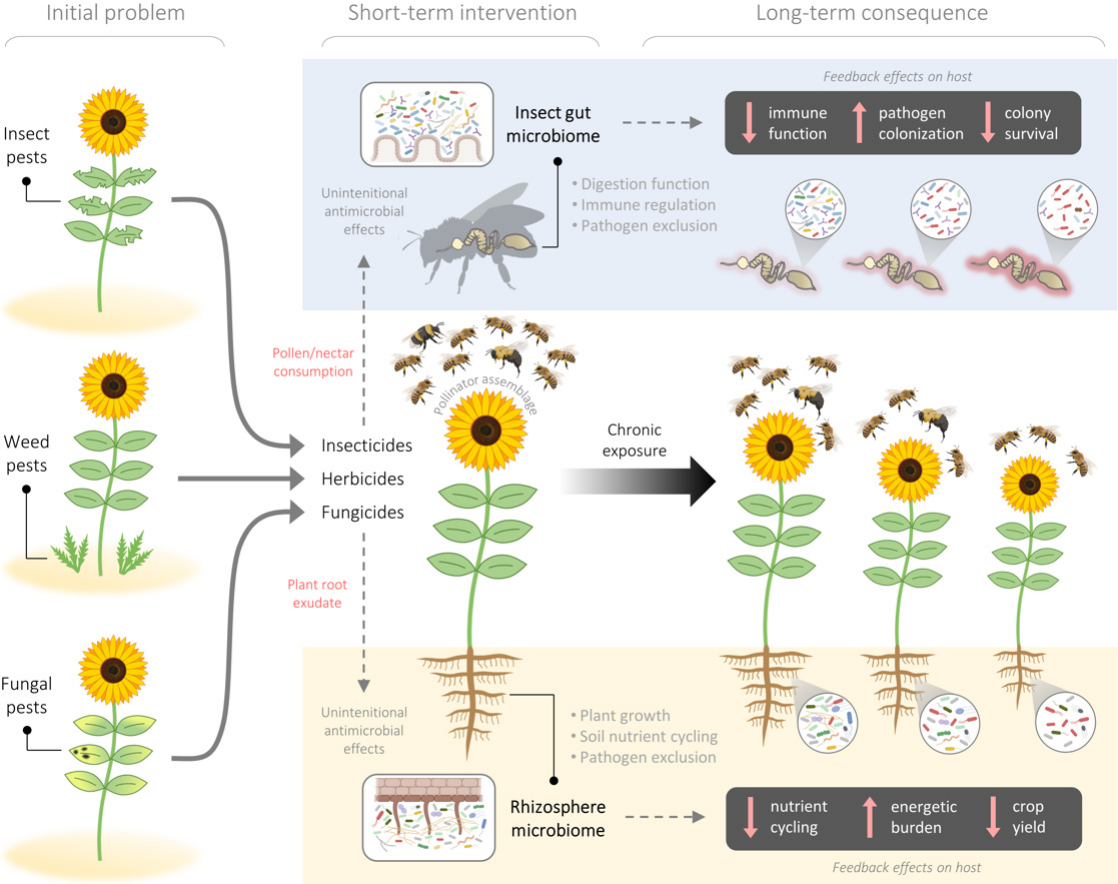
Schematic diagram illustrating pesticide-mediated microbiome effects on plants and insect pollinators. Insecticides, herbicides, and fungicides are commonly used to prevent crop diseases and minimize interference of crop pests in agriculture. These chemicals are widely popular for their perceptible benefits to crop health and yields over the short term. However, their unintentional antimicrobial effects can deteriorate the health-promoting microbial communities associated with plants and pollinators via chronic exposure through plant root exudate and pollen consumption, respectively. Ultimately, the feedback effects on host species have the potential to reduce long-term crop yields (via depletion of plant-growth promoting symbionts) and bee populations (via depletion of immune-regulating and pathogen excluding symbionts).

An analogous scenario could be the case for the effects of pesticides, which have revolutionized the agricultural industry (e.g., through minimizing crop loss to pest species) but may pose serious risks to wildlife metabolic health and long-term environmental sustainability. Previous reports have exhaustively described the consequential physiological effects of pesticides on off-target plant and animal tissue^[21-24]^. In this review, we detail the current knowledge relating to the important non-canonical mechanisms by which certain pesticides can obstruct plant and pollinator health via off-target interactions with the host microbiome [Figure 1]. Specific attention will be given to managed western honey bees (*A. mellifera*) on the basis of their proclivity to encounter pesticides, their unsustainable colony loss over the past decade, and their importance to agriculture and global food security.

## THE REGULATORY DILEMMA OF “NON-ANTIMICROBIAL” PESTICIDES

The Environmental Protection Agency (EPA) of the United States broadly classifies pesticides to be any chemical compound utilized for the purpose of killing crop pests that interfere with agricultural production - most commonly referring to that of herbicides, insecticides, and fungicides. A longstanding issue surrounding the use of pesticides, however, is the off-target deleterious effects they can have on a broad range of species found in terrestrial and aquatic ecosystems. In efforts to make sound regulatory judgements about these risks, authoritative bodies worldwide have attempted to implement minimum data and safety information requirements in relation to a given pesticide product’s potential for causing unreasonable adverse effects. For example, the EPA’s regulation “Data Requirements for Registration” (issued in 1984 under title 40, part 158 of the Code of Federal Regulations) specifies that risk assessments for the registration of new pesticides must evaluate the Ecological Risks, Human Health Risks, and Environmental Accumulation Risks^[25]^. It is important to note, however, that compliance with regard to ecological assessments extends only to “non-target plants, fish, and wildlife species” without any legislative guidance provided for microorganisms.

Only recently in 2013 did the EPA promulgate the final rules on data requirements (revised part 158 W) to provide distinct jurisdiction for “antimicrobial” and “non-antimicrobial” pesticides under the Federal Insecticide, Fungicide, and Rodenticide Act (FIFRA). Many pesticides with potential antimicrobial properties are nonetheless still registered as “non-antimicrobial” products (and are thus regulated under conventional mandates) as the result of mutually exclusive categorization schemas and a long list of exemptions. For example, an “antimicrobial pesticide” is defined under section 2 (mm) of FIFRA as any pesticide designed to disinfect, sanitize, reduce, or mitigate the growth of bacteria, viruses, fungi, protozoa, algae, or slime mold^[26]^. In nearly all cases, though, these criteria are nullified by the presence of additional claims (e.g., herbicidal or insecticidal properties), which result in the product (e.g., herbicides and insecticides) being classified as a “non-antimicrobial pesticide”. Perplexingly, agricultural fungicides are also considered a type of “non-antimicrobial pesticide” despite their registered intent as antimicrobial chemicals targeting fungal species. Legislative loopholes in classification such as these present a major concern as they obscure scientific communication and largely ignore the potential health hazards that common agrochemicals pose on plants and animals through interactions with their host-associated microbial communities.

## DISENTANGLING HOW PESTICIDES DAMAGE HOST MICROBIOMES

**Figure 2:**
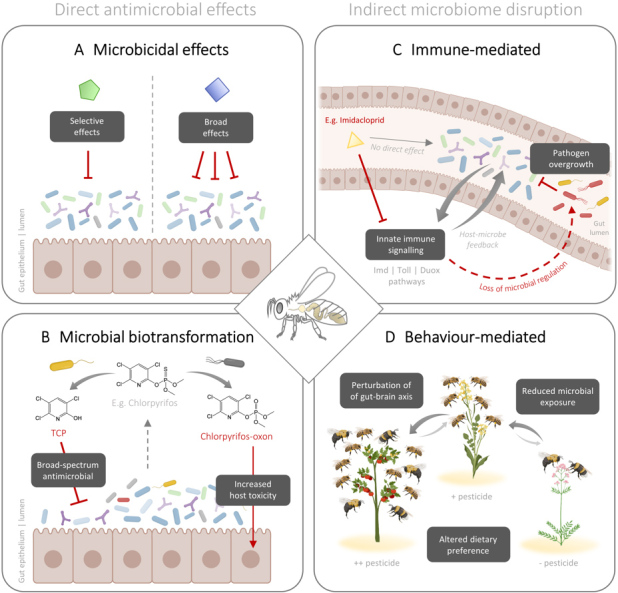
Direct and indirect mechanisms through which “non-antimicrobial” pesticides deteriorate bee-associated microbial communities. The panels on the left (A, B) highlight how both the parent compound and breakdown metabolites of pesticides can cause direct harm to microbial cells. The panels on the right (C, D) highlight how pesticides can alter microbial homeostasis through modulating host immune gene expression and behaviour in honey bees.

It is foreseeable that any chemical, in great enough quantity, could impede cellular biological function. Thus, the peak concentration and type of exposure (e.g., acute or chronic), as well as dose-dependent effects, are important considerations when evaluating the off-target antimicrobial effects of pesticides. Discussion in this review will accordingly focus on the antimicrobial mechanisms of insecticides, herbicides, and fungicides at environmentally realistic exposures. A brief in-text summary is provided for each of the relevant mechanisms shown in Figure 2, whereas a list of known interactions is reported in Table 1.

**Table 1:** Antimicrobial effects of common pesticides on plant- and pollinator-associated microbiomes

**Pesticide class**	**Pesticide name**	**Host**	**Effect on host-associated microbiome**	**Ref**
Insecticides	Heptachlor	Plants	Growth inhibition for ~92% of Gram-positive strains tested with no effect on any Gram-negative strains tested	[27]
	DDT	Plants	Decrease in active soil bacterial biomass by ~60% and an increase in fungal biomass by ~93%	[28]
	Carbaryl	Bees	Decreased total gut bacterial loads by ~90% (enumerated via qPCR) alongside a compositional depletion of Orbales at the order level	[29]
	Clothianidin	Bees	Gut region-specific signatures of dysbiosis in bacterial communities after 28-day exposure	[30]
	Imidacloprid	Bees	No effect on honey bee gut microbiota after 5-day of exposure and no effect on the growth of 16 honey bee-derived bacterial strains in pure culture	[31]
		Plants	Species-specific inhibition of ammonia-oxidizing archaea and ammonia-oxidizing bacteria	[32]
		Plants	Dose- and duration-dependent effects on diversity metrics of rice crop rhizosphere microbiome	[33]
		Plants	Reduction in culturable fungi by ~37%, coupled with the decrease in β-glycosidase, fluorescein diacetate hydrolase, acid phosphatase and urease enzymatic activities	[33]
	Imidacloprid and thiacloprid	Bees	Time-dependent effects on bacterial and fungal alpha diversity in honey bees during 35-day exposure	[34]
	Thiacloprid	Plants	Thiacloprid degradation by N_2_-fixing bacterium *Microvirga flocculans* produces breakdown metabolites that feedback to inhibit the growth	[35]
	Nitenpyram	Bees	Near-complete clearance of the symbiont *Gillimella* spp. in honey bees after 14-day exposure	[36]
Herbicide	2,4-Dichlorophenoxyacetic acid	Plants	Reduction of *nod* gene expression by ~32% in *Sinorhizobium meliloti*, ultimately affecting nitrogen fixation and plant hormone signaling	[37]
	Atrazine	Plants	Inhibited germination and ~80% reduction in radial growth of fungal symbiont *Trichoderma atroviride*	[38]
	Glyphosate	Bees	Reduction of symbiotic *Snodgrassella alvi *alongside the concurrent rise of entomopathogenic *Serratia marcescens* in honey bees	[39]
	Glyphosate	Plants	Increased prevalence of root-rot inducing *Fusarium* spp.	[40]
Fungicides	Carbendazim and Hexaconazole	Plants	Dose-dependent inhibition of plant-growth promoting *Pseudomonas* spp.	[41]
	Azoxystrobin and Chlorothalonil	Plants	Inhibited radial growth on agar by ~50% for the biocontrol fungus, *Fusarium oxysporum* CS-20	[42]
	Chlorothalonil	Bees	Altered structure of gut bacterial communities alongside predicted functional changes to carbohydrate metabolism after 6-week exposure	[43]
	Pristine (boscalid and pyraclostrobin mixture)	Bees	Dose-dependent compositional changes in the relative abundance of *Gilliamella* spp. and *Lactobacillus* Firm-4/Firm-5 members after 21-day exposure	[44]

DDT: Dichlorodiphenyltrichloroethane.

### Direct antimicrobial effects and lessons learned from legacy insecticides

The bulk of pesticide applications almost invariably reaches the soil, facilitating direct interaction with soil microbes [Figure 2A]. As a result of this intuitive linkage, some of the earliest evidence of pesticides exhibiting antimicrobial properties comes from studies on the soil microbiome and legacy organochlorine (OCL) insecticides. By design, OCLs target the nervous system of insects by binding to the GABA_A_ site of the gamma-aminobutyric acid (GABA) chloride ionophore complex, which ultimately causes paralysis and/or death via dysregulation of nerve cell membrane polarization. GABA is notably the most common inhibitory neurotransmitter in both vertebrate and invertebrate systems, and is especially crucial to honey bee foraging and grooming^[45]^. *In situ* investigations on OCLs demonstrate they can also have strong inhibitory effects on microbial growth and overall metabolic activities at the community level in soil^[28,46]^. Data from *in vitro* culture-based studies on hundreds of soil bacterial isolates confirm these effects, showing inhibition of ~74%-100% of the tested Gram-positive strains when exposed to field-realistic concentrations of γ-hexachlorocyclohexane, bandane, chlordane, heptachlor, and other OCLs^[27,47]^. Dichlorodiphenyltrichloroethane (DDT) is another well-known OCL with potent antibacterial properties, and under field conditions causes a ~60% decrease in active soil bacterial biomass and a ~93% increase in fungal biomass^[28] ^- the latter of which likely represents an indirect response to the former via reduced competition, rather than a stimulatory response to DDT since OCLs are notoriously recalcitrant to breakdown.

Importantly, the mechanism by which OCLs exert their differential antimicrobial effects has long been assumed to be through non-specific physicochemical disruption of (primarily Gram-positive) membrane-associated processes (e.g., ionic transport, electron transport, cell wall biosynthesis), ultimately leading to cell lysis and loss of viability^[48]^. That is, the antimicrobial effects of OCLs were thought to be random and independent from their designed functions of inhibiting insect GABAergic signalling. Recent evidence, however, suggests that GABA signalling (beyond its recognized role of neurotransmission in animals) plays a major role in cross-kingdom chemical communication and quorum sensing events that actively regulate bacterial-archaeal-fungal community structure^[49]^. GABA has also been found to represent an essential bacterial nutrient in certain Gram-positive bacteria, such as the recently identified human gut isolate, *Candidatus* “Evtepia gabavorous”^[50]^. Taken together, this suggests that the antimicrobial effects of OCLs are not random and may in fact be related to their inhibitory effects on GABAergic signalling. While OCLs are now banned in most countries due to their environmental persistence (facilitated at least in part by inhibition of their own bioremediation^[48]^) and association with a broad range of other wildlife health concerns^[51]^, they provide an exemplary account of host-microbiome interconnectedness and how intentional insecticidal properties can directly translate into unintentional microbicidal properties [Figure 1].

Other, perhaps more obvious examples of pesticides with antimicrobial properties are those of fungicides (or simply antifungals when considered beyond their agricultural usage). Pollinating insects are exposed to especially high levels of fungicides since they are considered “bee-safe” (in terms of acute toxicity) and are typically applied during periods of peak pollen bloom to prevent the growth of crop disease-causing fungal pathogens^[52]^. Perhaps unsurprisingly, a two-year study in managed honey bees showed that in-hive fungicide contamination was strongly associated with reduced overall fungal concentration and genus-level fungal diversity in beebread^[53] ^- the main dietary staple of honey bees which consists mostly of collected pollen. This is important since mounting evidence suggests that fungicides are negatively associated with pollinator health despite lacking signs of acute toxicity^[53-55]^, and that reductions in beneficial fungi are associated with poorer pollinator nutrition and increased susceptibility to fungal disease (e.g., Chalkbrood caused by *Ascosphaera apis*)^[56]^. Moreover, certain fungal steroids (produced by *Zygosaccharomyces* spp.) have recently been identified as essential to the development of stingless bees, which otherwise fail to pupate in their absence^[57]^. These findings potentially explain why group G fungicides (targeting sterol-biosynthesis) were disproportionately associated with colony loss in a large-scale study on migratory honey bee operations in the United States^[58]^.

Less intuitively, a broad range of fungicides (e.g., azoxystrobin, chlorothalonil, propamocarb, and propiconazole) can also negatively influence bacterial communities found in association with bees^[43,59,60]^. It is, however, difficult to ascertain the mode of action since the findings are correlative in nature. Based on the fact that supplementation of beneficial fungi in bees (e.g., *Aureobasidium melanogenum*) can increase bacterial community abundance^[61]^, it could be reasoned that a reduction in beneficial fungi (e.g., in response to fungicide exposure) may also have a negative effect on bacterial loads. Evidence from plants and rodent models supports the notion that fungi can play a major role in mediating community assembly within the rhizosphere microbiome^[62]^ and animal gut microbiome^[63]^, respectively. Fungicide-induced changes in bacterial communities could thus simply be the result of destabilized fungal-bacterial metabolic networks. Nonetheless, azole-based fungicides possess well established antibacterial properties^[64]^ and Khan *et al.*^[41]^ recently demonstrated *in vitro* that the two disjunct fungicides, hexaconazole and carbendazim (targeting sterol biosynthesis and microtubule assembly processes, respectively), could both exert direct bactericidal effects against plant growth-promoting *Pseudomonas* spp. in a dose-dependent manner - the mechanisms, however, have not yet been elucidated. Altogether, the current literature suggests that fungicides can directly disrupt host-associated microbiomes in multifaceted ways and that these off-target effects (which functionally diminish plant and animal health) are vastly understudied.

Lastly, glyphosate (commonly known as “RoundUp”) is the most popular herbicide used worldwide for weed control but has been associated with extensive disruption to plant and animal microbiomes^[65]^. These effects are explained by the fact that glyphosate targets the 5-enolpyruvyl-shikimate-3-phosphate synthase (EPSPS) enzyme used in the shikimate pathway (a central metabolic route affecting many adaptive processes^[15]^) of plants, bacteria, archaea, fungi, and some protozoa. Animals notably do not possess this pathway and thus glyphosate should theoretically demonstrate low toxicity towards them. In honey bees, however, Motta *et al.*^[66]^ demonstrated that glyphosate exposure results in dose-dependent, microbiome-mediated toxicity and reduced survival during infection with the Gram-negative opportunistic entomopathogen, *Serratia marcescens*. Data from *in vitro* studies support that glyphosate [rather than its breakdown metabolite aminomethylphosphonic acid (AMPA)] is the responsible factor involved and can exert differential antimicrobial properties on the basis of EPSPS class I (sensitive) and II (resistant) binding affinities^[67]^. The honey bee symbiont, *Snodgrassella alvi*, encoding a class I-type EPSPS has been reported to be consistently lower in abundance during exposure to glyphosate^[39]^. Together with evidence of *S. alvi*-mediated immunoregulatory roles^[68]^, this could potentially strengthen the otherwise somewhat obscure linkages between field-realistic glyphosate exposure and apparent susceptibility of honey bees to viral (Deformed wing virus) and fungal (*Nosema ceranae*) pathogens^[69]^.

Glyphosate can similarly increase the prevalence of root rot-inducing *Fusarium* spp. in the plant rhizosphere microbiome by inhibiting plant symbionts which otherwise antagonize the growth of the pathogen^[40]^. Moreover, the directly antifungal effects of glyphosate can impair mycorrhizal colonization and alter plant-soil nutrient cycling dynamics^[70] ^- an effect that can lead to long-term stunting of plant growth and a gradual reduction of crop yields^[71]^. While some studies contest the microbiome-mediated stunting effects of glyphosate^[72,73]^, a meta-analysis on the topic suggests that the phenomenon is dependent on soil pH differences^[74]^, which may govern the microbial degradation rates of glyphosate to its inactivate metabolite, AMPA. Collectively, the current literature indicates that glyphosate (and many other types of herbicides^[75]^) can directly exert unintentional antimicrobial effects on plant- and animal-associated microbial communities, and that these changes can consequently impair host developmental processes and disease resistance.

### Indirect antimicrobial effects via host-mediated immune dysregulation

Neonicotinoid-based insecticides, which induce neurotoxic effects via selective inhibition of insect nicotinic acetylcholine receptors, are widely popular as a result of their very low toxicity towards humans, but have faced much scrutiny with regard to their controversial association with declining pollinator populations^[76]^. One such topic of controversy is the administration of neonicotinoids, which are designed as “systemic pesticides” intended for uptake and distribution within plant tissue - the goal being to maximize target pest exposure while minimizing environmental contamination. However, neonicotinoids also accumulate in the plant root exudate and pollen (in the case of angiosperm plants)^[77]^, meaning that the rhizosphere microbiome and the gut microbiome of pollinating insects (via oral consumption of pollen) are the primary recipients of chronic off-target exposure. Nonetheless, studies on the antimicrobial effects of neonicotinoids have been inconsistent across the literature.

In considering the effects of imidacloprid (a common neonicotinoid) on the honey bee microbiome, Raymann *et al.*^[31]^ elegantly demonstrated that there were no obvious impacts on genus-level bacterial diversity metrics (measured via 16S rRNA gene sequencing) after five days of in-hive exposure. In the same study, *in vitro *exposure of 16 honey bee gut-derived bacterial isolates showed essentially no sensitivity (nor degradation ability) of the strains towards imidacloprid in mono- or mixed-culture experiments. Despite these seemingly conclusive findings, a follow-up study testing longer exposure durations (as would be expected from chronic hive contamination under realistic situations^[78]^) found that imidacloprid (and thiacloprid) exerted a time-dependent decrease in both bacterial and fungal community alpha diversity, with the most significant changes occurring after five weeks^[34]^. Empirical evidence also suggests that clothianidin^[30]^, nitenpyram^[36]^, thiamethoxam^[79]^, and other types of neonicotinoids^[80]^ can exert bee microbiome-disrupting side effects during longer periods of chronic exposure, although the responsible mechanism remains unclear.

One explanation could be that the immunosuppressive effects of neonicotinoids (thought to be at the root of globally declining populations of bees, fish, amphibians, bats, and birds^[81]^) act to reduce host-mediated selective pressures on microbial communities. This notion is supported by the fact that antimicrobial peptides (AMPs) and other effector molecules produced by the insect innate immune system (e.g., Imd, Toll, and DUOX pathways^[82,83]^) possess crucial microbiome-shaping properties via their differential activities against phylogenetically distinct microbial lineages. For example, the honey bee AMP, apidaecin, demonstrates magnitudes lower activity against Gram-negative symbionts (e.g., *G. apicola *and *Snodgrassella alvi *found abundantly in healthy bees) compared with Gram-negative opportunistic pathogens (e.g., *Escherichia coli*)^[84]^. A noteworthy point to highlight is that host-adapted symbionts possess unique functions, that in return, help shape innate immune signaling^[68]^. That is, the persistence of host-adapted microbial communities is a bidirectional process, and thus this may functionally explain how immune dysregulation by neonicotinoids could (indirectly) result in a loss of microbial diversity over time under realistic scenarios of chronic exposure. It is also foreseeable how these effects could exacerbate the loss of microbial diversity during concurrent exposure to chemicals that do possess antimicrobial capacities, such is the case for antibiotics^[85]^ and fungicides^[86] ^- the latter of which is consistent with results showing a near doubling of the apparent honey bee mortality risk over a four-month period during neonicotinoid co-exposure^[78]^. Collectively, the current literature suggests that the microbiome-disrupting effects of pesticides are not always as clear as direct inhibition, and in the case of neonicotinoids in bees, appear to be mediated indirectly via host immune dysregulation [Figure 2C], and at concentrations not otherwise directly toxic to bee physiology.

### Biotransformation-dependent antimicrobial effects

The potential direct antimicrobial effects of neonicotinoids cannot be ruled out based on bee microbiome studies alone. Nicotine represents a plant-produced signaling molecule known to interact with soil bacteria and fungi^[87]^, and thus neonicotinoids (i.e., structurally derived from nicotine) could foreseeably have a higher probability of interacting with plant-associated microbial communities. In the case of rice (*Oryza sativa*) crops, imidacloprid has been found to decrease diversity metrics in the rhizosphere microbiome, with impact severity shown to be both dose- and duration-dependent^[33]^. Thus, this could potentially imply an indirect plant-defense mediated response (i.e., similar to bees) given that imidacloprid (and thiamethoxam) can significantly reduce plant immune-related gene expression^[88]^. Nonetheless, laboratory studies on sandy soils (which deconvolute the potentially confounding variables of plant host-mediated responses on the rhizosphere microbiome) instead suggest that neonicotinoids can have a direct inhibitory effect on nitrifying organisms^[89]^, specifically reducing that of ammonia-oxidizing archaea and bacteria in a species-specific manner^[32]^. These findings are supported by other soil experiments also showing a dose-dependent decrease in important soil enzymatic activities (e.g., β-glycosidase, fluorescein diacetate hydrolase, acid phosphatase and urease) alongside a ~37% reduction in culturable fungi after 30 days of imidacloprid exposure^[33]^. Given that neonicotinoids can persist in soil for > 1000 days in some cases^[90]^, these effects could foreseeably disrupt long-term microbial homeostasis.

Overall, very little mechanistic work has been done to understand the antimicrobial effects of neonicotinoids, with the bulk of literature focusing on *in vitro* studies of soil isolates with neonicotinoid-degrading properties. Interestingly, many of these isolates are nitrifying or N2-fixing bacteria such as thiacloprid-degrading *Microvirga flocculans*^[35]^, thiamethoxam-degrading *Ensifer adhaerens*^[91]^, imidacloprid-degrading *Pseudomonas putida*^[92]^. Moreover, in each of these cases, as well in fungal degradation of imidacloprid by *Aspergillus terreus*^[93]^, the breakdown is directly coupled with growth impairment of the metabolizing strain - suggesting that neonicotinoid metabolites (e.g., nitroso-, guanidine-, and urea^[92]^) are the responsible antimicrobial factors involved, rather than the parent compounds. Other examples exist with at least 40 neonicotinoid-degrading isolates identified so far, mostly from soil or water environments (see review^[94]^). Notably, the preferential impact of neonicotinoids on plant growth-promoting N_2_-fixing bacteria appears to have long-term adverse outcomes in chickpea and soybean crop yields^[95,96]^, although data from corn crops show inconsistencies^[97]^. While these differences could potentially be attributable to variation in soil parameters (e.g., organic matter, pH, temperature, *etc.*) known to influence the behaviour of neonicotinoids^[98,99]^, there is, overall, a lack of literature on the topic and further studies are needed before any solid conclusions can be drawn.

Unlike animals, which rely on their diet for nitrogen via protein consumption, plants absorb nitrogen through their roots, often with the help of their associated microbiomes. This might explain why honey bee-associated microbial communities cannot degrade neonicotinoids and appear to be largely unaffected by their presence in culture^[31]^. It is nonetheless interesting to consider how insect-mediated detoxification of neonicotinoids (which also produces neonicotinoid metabolites, prior to excretion via the fecal-route^[100]^) could have insidious effects on the gut microbiome through activating the antimicrobial effects of these compounds. This has yet to be tested and would be a worthy direction for future studies. To note as well, microbial biotransformation-dependent toxicity of pesticides is not a unique process to neonicotinoids (see Table 1). Symbiont-mediated degradation of chlorpyrifos (a common organophosphate insecticide) in *Drosophila melanogaster*, for example, exerts pleiotropic effects by producing two metabolites - chlorpyrifos-oxon (with 10- to 100-fold higher insecticidal activity towards the host) and 3,5,6-trichloro-2-pyridinol (with potent antimicrobial effects on the gut microbiome)^[101] ^[Figure 2B]. Further, in pest insects such as the diamondback moth, alydid stinkbug, and crucifer root maggot, the degradation of various pesticides by microbial symbionts can infer insecticide resistance^[102-105]^. Together, these findings provide a basis to speculate on how “biopesticides” (i.e., microorganisms used for pest control) could be favorably utilized in tandem with pesticides to increase their insecticidal properties by *in vivo* biotransformation to more toxic metabolites - an approach that has been proposed in several emerging microbiome management strategies for agroecosystems^[106]^.

Overall, the discussed material cumulatively highlights three major points: (1) plant- and animal-associated microbiomes intrinsically differ in their sensitivity towards certain pesticides; (2) microbiome-mediated biotransformation of pesticides can produce antimicrobial metabolites from otherwise non-antimicrobial parent compounds; and (3) strategic modulation of host-associated microbiomes in agricultural systems has potential to offset adverse pesticide interactions.

### Behaviour-mediated antimicrobial effects

The antimicrobial effect of pesticides on pollinating insects can be amplified to a systems level through indirect effects on foraging and other social behaviours [Figure 2D]. Using the honey bee (*A. mellifera)* as an example, individual worker bees, which number in the tens of thousands per colony, forage separately for pollen and nectar. However, their industry is not selfish - instead, they return their foraged goods to the hive where it is concentrated into honey and pollen stores and ultimately fed to developing larvae^[107]^. The social foraging and otherwise colony-oriented behaviour of worker bees thus naturally concentrates any trace environmental contaminants from afar into higher, localized concentrations that can then accumulate and in some cases reach toxic levels^[108]^. Honey bees and other eusocial insects with this type of centralized foraging are thus vulnerable to bioaccumulation of neonicotinoids and other applied contaminants. The direct effects of pesticides on honey bee physiology have been intensely investigated^[109]^ but less is known about the indirect effects that likely arise from pesticide-mediated microbiome disruption and the downstream effects that this dysbiosis can have on individual and social behaviour.

Unique among insects, honey bees (as well as bumble bees) possess a “core” gut microbiome structure^[110]^ consisting of 8-10 species clusters within the genera *Gilliamella*, *Snodgrassella*, *Bombella*, *Lactobacillus*, *Apilactobacillus*, and *Bombilactobacillus*^[111]^. This community is remarkably consistent across environments^[112]^, suggesting a strongly co-adapted symbiosis is crucial to the maintenance of bee health and immunity^[113]^. For honey bees and other social insects in which individual behaviour has become integrated into a whole, the composition of symbiotic gut microbes can influence not only the behaviour of individual insects but also the collective behaviour of entire societies in which they live^[114]^. The brain-gut-microbiome axis is one mechanism by which gut microbiomes can influence the individual performance and social behaviour of workers within the hive^[115]^. Perturbation of this axis via pesticide-mediated depletion of core microbiome members should therefore affect bee behaviour in predictable and potentially manageable ways. For example, antimicrobial effects on bee gut microbiomes may alter foraging behaviour via individual performance or dietary preference^[116]^, which when amplified across all individuals and colonies, can impact pollination services.

Interestingly, the learning performances of honey bees are differentially affected by imidacloprid according to the season^[117]^. Underpinning this phenomenon could be the substantial fluctuation in gut microbiome structure known to occur between winter and summer seasons^[118]^. There is ample opportunity for feedback between host and microbiome if, for example, initial changes to microbial communities then bias foraging preference to influence the plant-associated microbes the bees are exposed to. Subtle behavioural changes (i.e., otherwise not impacting host survival by itself) could thus hinder natural plant-pollinator microbial exchange processes and influence the long-term maintenance of co-adapted symbionts. Some remediation may be possible through the application of beneficial bacteria that off-set the dysbiosis inadvertently caused by the well-intended application of commercial antibiotics^[85]^ or pesticides^[119]^ to hives.

Given that bee social behaviour is highly coordinated, where the worker caste can specialize into behavioural subcastes that, besides foragers, include nurses, guards, hygienists, undertakers and scouts^[120]^, we expect any significant variation in the gut microbiome to affect the bee’s most fundamental behaviours, including recruitment, hygienic, defensive and appetitive behaviours - all of which are essential to colony’s eusocial structure^[121]^. For humans, communication along the brain-gut axis is mediated through immune mechanisms, elements of the nervous system and microbial metabolites that relay nutrition and health status from the gut to the brain^[122]^. For insects and bees in particular, the mechanics of this axis are less well defined, however, because social insects have evolved a particularly strong dependence upon gut symbionts^[6]^, there is a strong rationale for investigation of this topic in future studies.

## LINKAGES BETWEEN PESTICIDE EXPOSURE AND ANTIMICROBIAL RESISTANCE

Pesticides, like antibiotics, represent chemical stressors that can exert selective pressures on microbial communities. Mounting evidence suggests that the evolution of tolerance, resistance, and persistence^[123]^ towards pesticides may consequently impact microbial response to antibiotics through both generalizable and specific mechanisms^[124]^. This represents a major human health concern in relation to the rise of multidrug-resistant pathogens (or “superbugs”) and hospital-acquired infections that are increasingly difficult to treat.

One mechanism of overlapping resistance is through efflux pumps (e.g., SMR and MATE families in particular^[125]^), which are membrane-bound transporters that can export multiple toxic substrates out of the cell. For example, Kurenbach *et al.*^[126]^ found that pre-exposure of *E. coli* to two herbicides (glyphosate and dicamba) could significantly increase subsequent tolerance against two broad-spectrum antibiotics (chloramphenicol and kanamycin) via overexpression of the AcrAB efflux pump - an effect that failed to occur in the presence of efflux pump inhibitor Phe-Arg β-naphtylamide. Others have also reported that biocide usage selects for overexpression of efflux pumps^[127]^. Alternatively, a study on realistic co-exposure of 23 pesticides in *E. coli* demonstrated that streptomycin-resistance emerged rapidly via selected for mutations in *acrR* (encoding a transcriptional repressor that regulates *acrAB* expression) as well as biofilm, heat shock, oxidative stress defense, and carbon starvation genes^[128]^.

In the case that pesticides cannot be pumped out of the cell, intrinsic or acquired enzymatic functions can facilitate overlapping resistance to antibiotics. For example, the plasmid-encoded organophosphorus hydrolase (OPH) of insecticide-degrading *Bacillus* isolates (e.g., *B. cereus*, *B. firmus*, and *B. thuringiensis* strains from contaminated agricultural sites) can confer multidrug resistance by inactivating chloramphenical, monochrotophos, ampicillin, cefotaxime, streptomycin and tetracycline antibiotics^[129]^. The noteworthy point is that similar or identical OPHs have been found in *Pseudomonas*, *Flavobacterium*, *Sphingobium*, and *Agrobacterium* spp.^[130-132]^, with molecular evidence suggesting the response genes have probably evolved within the past 70 years^[133]^. This coincides directly with the introduction of organophosphate insecticides and the global rise of antibiotic resistance, and thus it is interesting to speculate how these processes have co-impacted the evolutionary trajectory of microbial life. Similar comparisons can be made for several oxidoreductases, transferases, and lyases in terms of conferring overlapping pesticide and antibiotic resistance properties^[124]^.

## FUTURE DIRECTIONS

Several important areas have been highlighted in this review that deserve future scrutiny. These are summarized below:

(1) Currently, regulatory oversight of agrochemical usage is inadequate and fails to address potential effects on ecosystem microbiomes which are in turn critical to environmental health. A reassessment of the legislative framework that governs the use of agrochemicals is urgently warranted.

(2) Agrochemical toxicity is generally defined as a construct of their direct and acute harmfulness towards plant and animal species, whereas their detrimental effects on plant- and animal-associated microbiomes are likely to have more subtle, accumulative consequences. Long-term studies of plant and animal health (including measurements of microbiome diversity, composition and activity) following exposure to agricultural compounds are required to allow balanced calculation of risk *vs.* benefit of agrochemical use.

(3) Microbial biotransformation of agrochemicals is understudied and requires urgent evaluation if we are to fully understand the impact of a given compound on the environment. Relatedly, bioremediation efforts need to account for potential adverse effects of breakdown metabolites on not only plant and animal physiology, but also their host-associated microbial communities.

(4) Antimicrobial resistance is a current global threat to health, and it is imperative that the role of agrochemical use in the development of antimicrobial resistance is fully studied and appreciated.

(5) The gut-microbiome-brain axis is an emerging field of interest with relevance to pesticide-neuroimmune interactions and merits particular attention in eusocial bee species, such as honey bees, which exhibit strong interdependencies on their gut microbiomes.

In addressing these issues as they relate to microbiome-mediated pesticide toxicity, proof of causality is an important factor to consider. Multi-omics technologies have massively improved our ability to identify taxonomic- and functional-based correlations of host-associated microbial communities in response to agrochemical exposures. However, demonstrating an association between pesticide exposure and microbiome change is not enough to authenticate an antimicrobial effect. As discussed in this review, it remains challenging (due to confounding host variables) to delineate between direct antimicrobial properties and indirect microbiome disrupting activities based on *in vivo* observation alone [Figure 2]. Indeed, culture-based interrogation of host-derived isolates *in vitro* can be extremely informative, although not all microbes are culturable and pesticide co-culture experiments with single strains are not adequately representative of the highly complex polymicrobial interactions that may occur under natural conditions (see Ref.^[134]^ for review on methodological challenges of pesticide risk assessment).

Collectively, this indicates a substantial need for model development of host-free rhizosphere and insect gut microbiome systems. Bioreactor models for the human gut microbiome already exist (e.g., benchtop “Robogut”^[135]^ and SHIME systems^[136]^) and are actively being used to decipher microbe-drug interactions relevant to human disease treatments^[137]^. Notably, these systems may be easily adapted for agricultural purposes as well, specifically allowing for high-throughput evaluation of microbe-pesticide interactions pertaining to honey bee health, commercial crop yield, and environmental health as a whole. Future development and testing of such models should be an immediate priority based on the fact that current pesticide risk assessment strategies could also benefit from their use by improving the veracity of new product safety claims prior to their release.

## OUTLOOK AND CONCLUDING REMARKS

We have outlined the multifaceted ways in which agricultural chemicals can disrupt microbial ecosystem function using examples from honey bees and crop plants. Perhaps one of the more alarming aspects of the current situation is the apparent weakness of regulatory policies, which are riddled with loopholes and largely ignore contemporary research findings on host microbiome-pesticide interactions.

There is a pressing need to reassess the use of agrochemical xenobiotics through the lens of microbial ecology and the concurrent or subsequent effects on host (animal and plant) physiology. This is not easy to do, since most microbial ecosystems are highly complex, and so much of the microbial world remains unstudied. However, tools to study microbiomes and their functions are now increasingly accessible, and should be exploited to study microbial ecosystem modulation by herbicides, insecticides and fungicides across all agricultural sectors as a matter of great urgency.

## DECLARATIONS

### Copyright

© The Author(s) 2022.
